# Material Hardship Among Custodial Grandparents and Grandchildren's Physical and Mental Health in COVID-19

**DOI:** 10.1093/geroni/igab046.1902

**Published:** 2021-12-17

**Authors:** Yanfeng Xu, Qianwei Zhao, Brittany Schuler, Sue Levkoff

**Affiliations:** 1 University of South Carolina, Columbia, South Carolina, United States; 2 Baylor University, Waco, Texas, United States; 3 Temple University, Temple University, Pennsylvania, United States

## Abstract

COVID-19 has increased economic hardship for many families, including custodial grandparent-headed families. We aim to examine latent classes of material hardship among custodial grandparent-headed families, to assess predictors associated with identified classes, and to investigate associations with grandchildren’s physical and mental health outcomes during COVID-19. Data was collected from a cross-sectional survey in June 2020. The sample comprised of 362 grandparents. Latent class analysis and logistic regression were conducted. Three latent classes of material hardship were identified: Class 1 (n = 232; 64.1%) low overall hardship with high medical hardship, class 2 (n = 52; 14.4%) moderate overall hardship with high utility hardship, and class 3 (n = 78; 21.5%) severe overall hardship. Factors, such as race, household income, labor force status, financial assistance status, and trigger events to raise grandchildren, were associated with class membership. Class 2 (OR = 0.19, p < 0.05) compared to Class 1 was significantly associated with grandchildren’s physical health. Our findings suggest that material hardship is heterogeneous among custodial grandparents during COVID-19, and children in households experiencing utility hardship have a higher risk for poorer physical health outcomes. Results highlight the needs to meet grandparents’ material needs and call for future research to examine the mechanism that explains the link between material hardship and grandchildren’s outcomes.

